# Assessment of test-retest reproducibility by [^18^F]Bavarostat for PET imaging of HDAC6

**DOI:** 10.1186/s13550-025-01268-w

**Published:** 2025-06-21

**Authors:** Mika Naganawa, Ming-Qiang Zheng, Jean-Dominique Gallezot, Robin Bonomi, Jiwei Gu, Hong Gao, Swanee Jacutin-Porte, Nabeel B. Nabulsi, Michel Koole, Koen Van Laere, David Matuskey, Yiyun Huang, Richard E. Carson

**Affiliations:** 1 Yale PET Center, Yale School of Medicine, PO Box 208408, New Haven, 06520-8048 USA; 2 Nuclear Medicine and Molecular Imaging, Department of Imaging & Pathology, KU Leuven, Leuven, Belgium

**Keywords:** Positron emission tomography, Test-retest reproducibility, Brain imaging, Human study, HDAC6

## Abstract

**Background:**

Histone deacetylase 6 (HDAC6) is an enzyme pivotal for gene regulation, influencing cellular pathways through protein deacetylation. HDAC6 is a potential therapeutic target in diseases such as cancer and neurodegenerative disorders. Koole et al. investigated brain binding of [^18^F]Bavarostat, an HDAC6 inhibitor, in healthy participants, revealing an absolute test-retest variability (aTRV) of 7.7% (*n* = 4) for the distribution volume (*V*_T_) with a 1-day interscan interval. This study aims to evaluate test-retest reproducibility with a more extended interscan interval.

**Results:**

Six participants (3 M/3F) underwent a test-retest scan, each lasting for 120 min using a 4-ring Biograph mCT PET/CT scanner. Arterial blood sampling and metabolite analysis were performed to derive the input function. The two scans were 28 ± 12 days apart (14–43 days, *n* = 6). Regional time-activity curves (TACs) were generated for 15 regions of interest (ROIs). Kinetic analysis of the 120-min TACs was performed using one-tissue and two-tissue compartment models (1TC, 2TC) and multilinear analysis-1 (MA1) to quantify *V*_T_ values and compute absolute test-retest variability (aTRV). The effects of scan duration (60 to 120 min) and MA1 t* setting on aTRV and bias were investigated. Careful analysis of the plasma HPLC data was needed since metabolites eluted close in time to the parent. The MA1 model (t* = 40 min) adequately described regional TACs and produced stable kinetic parameters with good agreement to 2TC (MA1 *V*_T_=0.98 × 2TC *V*_T_ + 0.48, bias: -0.1%) while 1TC underestimated *V*_T_ by 5.1%. Regional *V*_T_ values exhibited a relatively uniform pattern, highest in the amygdala and lowest in the centrum semiovale. Individual aTRV values ranged from 2 to 9%. Scan durations between 100 and 120 min provided the most consistent results, with minimal bias and acceptable aTRV across all tested t* values. Although a 90-minute scan with t*=10 or 20-minute balanced scan time and aTRV, optimal parameters varied by brain region. Smaller regions (e.g., amygdala) required longer scans to achieve reliable *V*_T_ quantification.

**Conclusions:**

The test-retest variability of [^18^F]Bavarostat *V*_T_ values demonstrated favorable results for a one-month scan interval, comparable to the reported values.

**Supplementary Information:**

The online version contains supplementary material available at 10.1186/s13550-025-01268-w.

## Introduction

Epigenetics plays a crucial role in regulating gene expression without altering the DNA sequence itself. A key mechanism involves histone modifications, including acetylation by histone acetyltransferases and deacetylation by histone deacetylases (HDACs). HDACs remove acetyl groups from histone proteins, impacting how tightly DNA is packaged within the nucleus and ultimately affecting gene activity.

Among the HDAC family, HDAC6 has garnered significant interest due to its involvement in various cellular pathways, particularly within the brain. Notably, HDAC6 is unique as it primarily deacetylates proteins in the cytoplasm, including those critical for microtubule assembly and function [1]. Researchers are exploring its potential role in the development and progression of various diseases [2, 3] including neurodegenerative diseases, such as Alzheimer’s disease [4, 5, 6], Parkinson’s disease [7, 8], and amyotrophic lateral sclerosis [9], psychiatric diseases [10, 11, 12], and cancer [13, 14, 15]. Understanding how in vivo HDAC6 expression changes within the human brain could provide valuable insights.

[^18^F]Bavarostat (also known as [^18^F]EKZ-001) has emerged as a promising radiotracer for positron emission tomography (PET) imaging [16, 17, 18]. This tracer was developed with > 117 times stronger affinity for HDAC6 over HDAC class I. This radiotracer possesses several advantageous properties for PET imaging, including excellent penetration into the brain and high selectivity for HDAC6, allowing visualization of its expression in the brain. A previous study by Koole et al. [18] revealed that the two-tissue compartment model and Logan graphical analysis were the preferred models, with stable *V*_T_ estimation achievable in a ‘coffee break’ protocol. This protocol, which includes an initial 60-minute scan followed by a second scan from 90 to 120 min, provided similar test-retest variability (TRV) compared to a continuous 120-minute scan.

Before [^18^F]Bavarostat can be confidently employed in wider research settings, a crucial aspect needs to be thoroughly evaluated: its test-retest reproducibility. This concept refers to the consistency of measurements obtained across multiple scans of the same individual. Test-retest reproducibility is critical for ensuring that a biomarker accurately reflects underlying biological processes. While the goal of test-retest studies is often to optimize methodology and minimize variation introduced by quantification techniques, some degree of biological variability is expected. This is particularly relevant for targets such as epigenetic regulators, which may fluctuate over time due to various biological or environmental factors. The presence of such variability does not undermine the utility of test-retest designs. Instead, it highlights the need to determine whether observed variability remains within an acceptable range for reliable interpretation across clinically relevant timescales. Test-retest variability can be influenced by several factors. General factors such as scanner performance consistency and uncorrected head motion affect all tracers. Other contributing factors, such as intrinsic statistical noise due to limited radiotracer counts and image processing techniques, also contribute variability. However, the main focus of a test-retest variability study is on tracer-specific factors, particularly physiological variation and the effects of the chosen kinetic modeling method. These factors are critical for understanding the test-retest variability specific to [^18^F]Bavarostat. By establishing robust test-retest reproducibility, researchers can be confident that the observed changes in the biomarker are not merely artifacts of the measurement process but rather reflect genuine biological phenomena, such as disease progression or treatment response. Moreover, test-retest results provide valuable information for future study design, such as enabling power analyses in longitudinal drug studies where intra-individual variability naturally contributes to changes from baseline to post-drug scans.

While [^18^F]Bavarostat exhibits great promise for measuring HDAC6 in the human brain, a single human study by Koole et al. [18] has so far investigated its test-retest reproducibility. This initial study employed a one-day interscan interval and reported encouraging results with an absolute test-retest variability (aTRV) of 7.7% (*n* = 4) for distribution volume (*V*_T_). However, the impact of longer interscan intervals, which are likely to be more relevant in some research settings, remains unexplored. This study aims to bridge this gap by evaluating the test-retest reproducibility of [^18^F]Bavarostat PET for quantifying HDAC6 in the human brain using a longer interscan interval compared to previous studies. The motivation for using a longer interval is to investigate whether an epigenetic regulator, which could vary over time due to multiple factors, remains stable over a longer period of time than a single day. Additionally, we investigate the influence of scan duration on test-retest reproducibility, providing further insights into optimizing [^18^F]Bavarostat PET protocols.

## Materials and methods

### Radiotracer synthesis

[^18^F]Bavarostat was synthesized as previously described, with modifications to produce the final dose for human injections [17, 18]. In brief, the trapped [^18^F]fluoride on SPE Chromafix 30-PS-HCO_3_ cartridge was washed by 1 mL of ethanol, eluted with 3–4 mg of phenol precursor, 4–5 mg of N, N-bis(2,6-diisopropylphenyl)-1-chloroimidazolium chloride (ClIm) and 3–5 mg of Ru-complex (CpRu(cod)Cl) in ethanol and the cartridge was rinsed with acetonitrile (MeCN) and dimethyl sulfoxide (DMSO) and the solutions were collected into the same reaction vial. The reaction vial was sealed and heated at 130 ^o^C for 30 min. After cooling, a mixture of THF/MeOH, aqueous hydroxylamine (NH_2_OH) and 5 M sodium hydroxide (NaOH) were added, and the reaction vial was stirred for 10 min. The solution was diluted with water and loaded onto an OASIS MAX SPE cartridge, and then purified by semi-preparative HPLC (XBridge, C18, 5 μm, 250 × 10 mm, mobile phase consists of 70% 190 proof USP ethanol and 30% 0.05 M USP sodium acetate (NaOAc), pH adjected with USP glacial acetic acid to 5.5–5.6, flow rate at 3 mL/min). The retention time of the product is between 18 and 22 min. A 1-min HPLC product fraction (3 mL) was collected, mixed with 6 mg of sodium ascorbate, and diluted with 20 mL of sterile saline containing 40 mg of sodium ascorbate. The resulted solution was passed through a 0.22 μm filter into a final dose vial ready for injection.

### Human subjects

A total of six healthy volunteers (3 males and 3 females, aged 49 ± 10 years; range, 30–58 years) were recruited and medically screened and cleared for this study. The study protocol was approved by the Yale Human Investigation Committee and the Yale University Radiation Safety Committee. All procedures adhered to federal guidelines and regulations of the United States, specifically those outlined in Title 45 Part 46 of the Code of Federal Regulations (45 CFR 46), aimed at safeguarding human research subjects. Written informed consent was obtained from all participants.

### Brain PET studies

#### PET imaging

Two 120-minute PET scans were acquired on separate days (test and retest) for seven healthy participants using a Biograph mCT PET/CT scanner (Siemens Medical Solutions, Knoxville, TN) following intravenous administration of [^18^F]Bavarostat. The [^18^F]Bavarostat bolus injection was delivered over 1 min using an automatic pump (Harvard PHD 22/2000, Harvard Apparatus Holliston, MA, USA).

The frame timing comprised 6 × 30 s, 3 × 1 min, 2 × 2 min, and 22 × 5 min with corrections for attenuation, normalization, scatter, randoms, and dead time using the MOLAR algorithm [19]. To minimize motion effects, event-by-event motion correction [19] was incorporated into the reconstruction process, utilizing measurements acquired by the Polaris Vicra sensor (NDI Systems, Waterloo, Canada) with reflectors mounted on a cap worn by each participant. The test and retest scans were conducted with an average interval of 28 ± 12 days (range 14–43 days, *n* = 6).

#### Magnetic resonance imaging

For co-registration with PET images, all participants underwent MR imaging (MRI) on a 3T whole-body scanner (Prisma; Siemens Medical Systems) equipped with a circularly polarized head coil. A 3D MPRAGE pulse sequence was employed with an echo time of 2.81 ms, a repetition time of 2530 ms, an inversion time of 1100 ms, and a flip angle of 7.

#### Measurement of arterial input function and free fraction in plasma

Blood samples were collected at discrete intervals during the experiment. Samples were manually drawn every 10 s from 0 to 90 s, every 15 s from 90 s to 3 min, and subsequently at 3.5, 5, 6.5, 8, 12, 15, 20, 25, 30, 45, 60, 75, 90, 105 and 120 min. Following collection, samples were centrifugated to separate plasma from whole blood, and these samples were analyzed using a calibrated well counter to quantify the radioactivity concentration.

Plasma analysis of radiotracer metabolism was conducted using samples collected at specific time points: 3-, 8-, 15-, 30-, 60-, 90-, and 120-minutes post-injection. The metabolite analysis utilized the column-switching HPLC method [20] to determine the parent fraction. The HPLC system was equipped with a capture column (2.1 × 20 mm) self-packed with Phenomenex Strata-X polymeric SPE sorbent and eluting with 30% MeCN in water at 2 mL/min for 4 min. The trapped activity in the capture column was then back flushed and eluted through an XBridge C18 analytical column (250 × 4.6 mm, 5 μm) with 30% MeCN in 0.1 M ammonium formate (v/v, pH = 6.4–6.6) at a flow rate of 1.65 mL/min. The parent fraction was determined as the ratio of the area under the parent peak to the total area of under the curve and fitted with a sigmoid function based on the inverted gamma function. Due to peak overlap with radiometabolites, the parent peak retention time was manually determined for each sample for a more precise calculation. The arterial plasma input function was then calculated as the product of the radioactivity concentration in the plasma and the interpolated parent fraction at each time point. An ultrafiltration-based method (Centrifree, Millipore) was used to determine the free fraction (*f*_P_) of the radiotracer in plasma.

#### Image registration and regions of interest

PET images were motion-corrected using frame-by-frame registration to a summed image (0–10 min post-injection) employing a mutual information algorithm (FSL-FLIRT). Subsequently, the summed PET image underwent co-registration with the participant’s T1-weighted MR image using a rigid registration. The T1-weighted MR image was further co-registered with the Automated Anatomical Labeling (AAL) template [21] in Montreal Neurological Institute (MNI) [22] space using a nonlinear transformation (Bioimage suite) [23]. Using the resulting composite transformations from template-to-PET space, regional time-activity curves (TACs) were obtained for regions of interest (ROIs) including the amygdala, anterior cingulate cortex, caudate, cerebellum, frontal cortex, globus pallidus, hippocampus, insular cortex, occipital cortex, parietal cortex, posterior cingulate cortex, putamen, temporal cortex, and thalamus, along with the centrum semiovale. TACs of gray matter ROIs were masked using segmentations derived from Computational Anatomy Toolbox (CAT12).

#### Quantitative analysis

Regional distribution volumes (*V*_T_) were computed using the one-tissue (1TC) and two-tissue (2TC) compartment models. Additionally, Multilinear Analysis 1 (MA1) was applied, exploring various starting time points: t*=10, 20,…, 60 min. The metabolite-corrected plasma activity served as input function for these analyses.

#### Test-retest Evaluation

The test-retest variability (TRV) reflects the magnitude and direction of the difference between parameter estimates obtained from the test and retest scans. It is expressed as the difference between the test and retest values divided by their average: (retest – test) / (test + retest) × 2. The mean of TRV indicates the presence of a systematic bias between the scans. The standard deviation of TRV, on the other hand, quantifies the variability in the difference between the two estimates. The absolute value of TRV (aTRV) is calculated as the absolute value of TRV. The mean aTRV can be interpreted similarly to the error associated with a single measurement, offering a straightforward assessment of reproducibility. The intraclass correlation coefficient (ICC) was used to evaluate reliability, calculated as (BSMSS-WSMSS)/(BSMSS + WSMSS), where BSMSS and WSMSS represent the between-subject and within-subject mean sum of squares, respectively.

#### Scan duration and t* effect on *V*_T_ and test-retest variability

The effect of scan duration and the choice of t* for MA1 on *V*_T_ and test-retest variability was evaluated. Tested scan durations ranged from 60 to 120 min with 10-minute increments and the t* settings varied between 10 and 60 min (*n* = 6 test-retest pairs). The aTRV for each combination of scan duration and t* setting was computed. The bias in *V*_T_ was assessed by comparing the estimated *V*_T_ for each scan duration and t* setting against the 2TC model with 120-minute scan duration. The mean aTRV and mean bias were obtained as the volume-weighted average across all brain regions.

## Results

### Radiotracer synthesis

The radiotracer was obtained with > 98% radiochemical and chemical purity in a total synthesis time of 100 min, and an average molar activity of 86.3 ± 57.4 GBq/µmol (*n* = 12) at the end of the synthesis.

### Tracer injection and scan parameters

The administered activity dose of [^18^F]Bavarostat was 131 ± 48 MBq and 150 ± 53 MBq at test and retest scans, respectively. The injected mass was 1.02 ± 0.36 µg (test) and 0.80 ± 0.35 µg (retest). Statistical analysis revealed no significant differences between the test and retest scans for these parameters (Wilcoxon signed-rank test, *P* > 0.5). The plasma free fraction *f*_P_ was 0.033 ± 0.010 (test) and 0.027 ± 0.011 (retest). No statistically significant difference was observed in *f*_P_ between the test and retest groups (Wilcoxon signed-rank test, *P* > 0.3). Injection and scan parameters are provided in Table 1.

### Plasma analysis

**Fig. 1:**
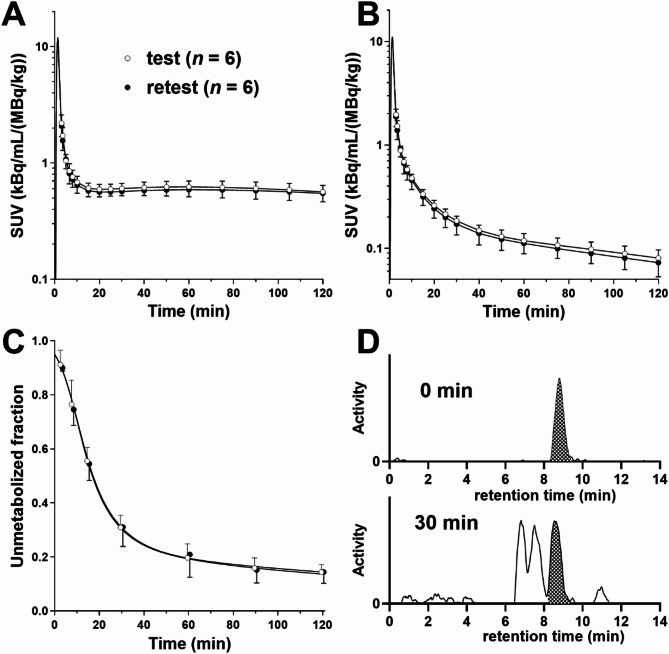
Mean ± SD of (**A**) total plasma activity, (**B**) metabolite-corrected plasma activity, and (**C**) parent fraction in plasma following administration of [^18^F]Bavarostat at the test (open circles, *n* = 6) and retest (closed circles, *n* = 6); **D**: Representative HPLC chromatograms from metabolite analysis of [^18^F]Bavarostat in plasma at 0- and 30-min post-injection. The filled peak corresponds to the parent compound

Plasma data and representative reverse-phase HPLC chromatograms at selected time points are displayed in Fig. 1. The parent fraction in plasma was determined and found to be comparable between the test and retest scans. The mean parent fractions at 30, 60, and 90 min after injection were 31 ± 5%, 20 ± 5%, and 16 ± 4% (test) and 31 ± 7%, 21 ± 8%, and 15 ± 5% (retest), respectively. Due to partial overlap of metabolites with the parent peak (Fig. 1D), a potential over- or underestimation of the parent fraction may occur.

### Brain distribution and kinetic analysis

**Fig. 2:**
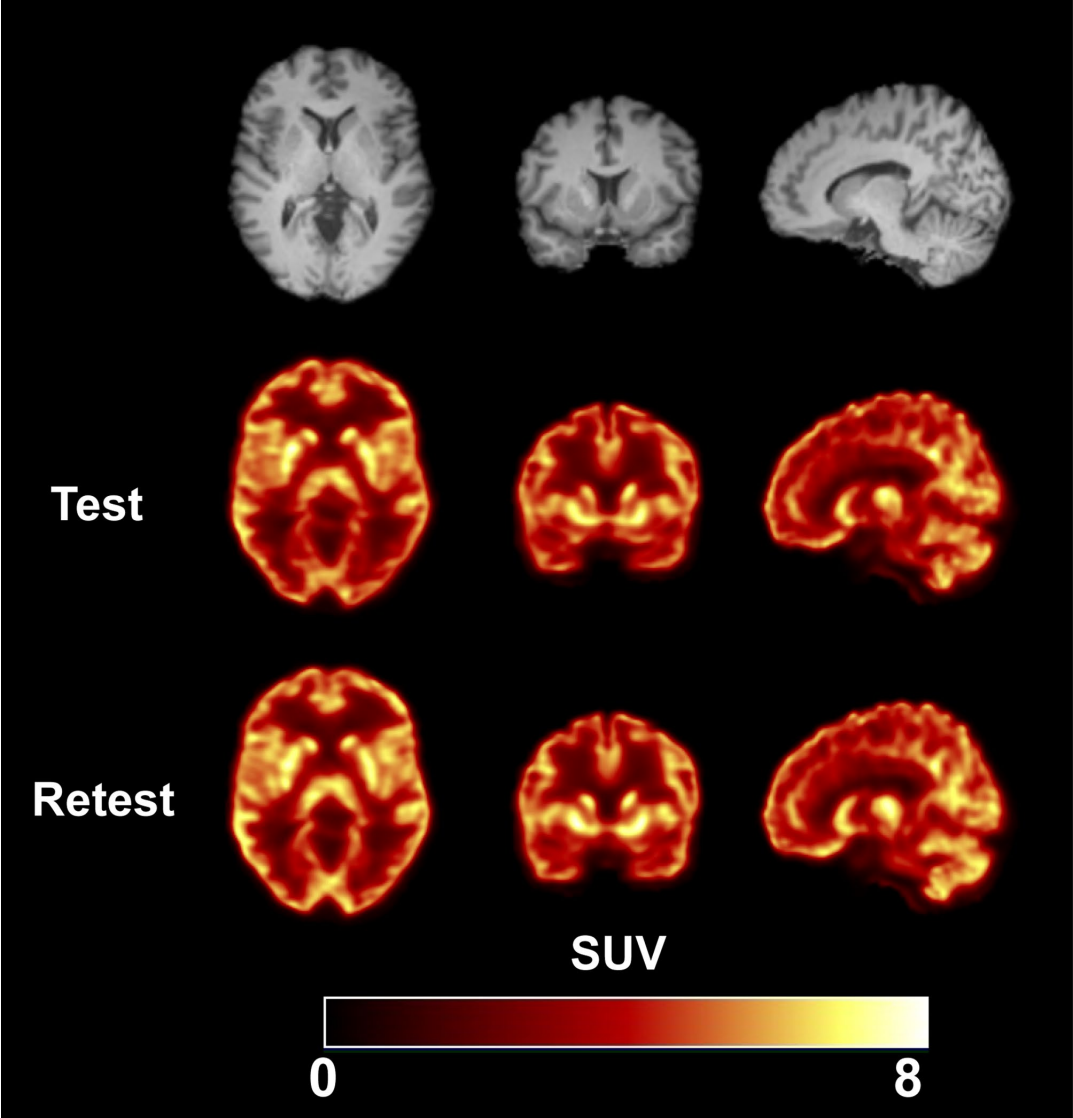
MR and co-registered PET SUV images (summed from 0–120 min of data) from test and retest scans in the same subject

**Fig. 3:**
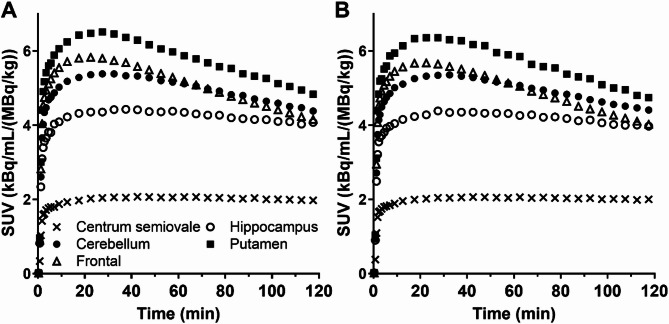
Mean regional time-activity curves from test (**A**) and retest (**B**) scans of [^18^F]Bavarostat (*n* = 6)

**Fig. 4:**
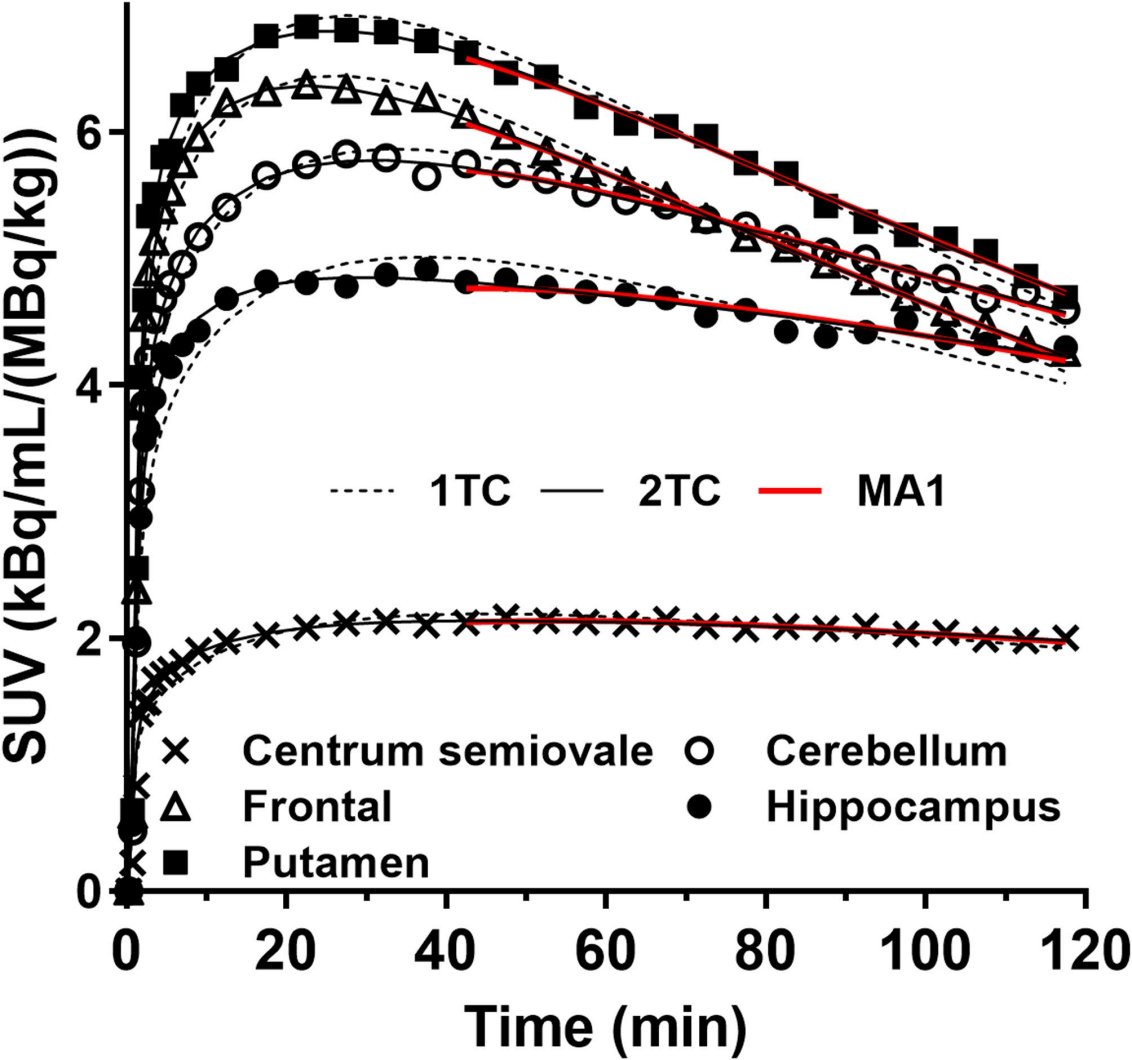
Time-activity curves in five ROIs with the 1TC (dashed lines), 2TC (solid black lines), and MA1 (t* = 40 min, solid red lines) fits. For each region, the symbols correspond to the measured regional activity

Representative PET images from the test and retest scans of [^18^F]Bavarostat in the same subject are shown in Fig. 2. The early and late images showed similar uptake distributions. Selected regional TACs are shown in Fig. 3. Standardized uptake value (SUV) peaked at approximately 30 min post injection in both scans, and TACs from the test and retest displayed high concordance. The 2TC model was statistically favored over the 1TC model for curve fitting in 94% of TACs, as determined by the *F*-test, where the *F*-statistic was greater than *F*(2,29) = 3.33 for *P* = 0.05. Additionally, comparison of the Akaike Information Criterion (AIC) values indicated the 2TC model was preferred in 95% of the 180 fits. The MA1 model (t* = 40 min) also demonstrated good fitting characteristics (Fig. 4).

1TC underestimated *V*_T_ compared to 2TC by 5.1% (volume-weighted mean across regions, $\:1\text{T}\text{C}\:{V}_{\text{T}}\:=\:0.90\:\times\:\:2\text{T}\text{C}\:{V}_{\text{T}}\:+\:1.14,\:{R}^{2}=0.96$). Regional differences were observed, with larger discrepancies in the amygdala (8%), hippocampus (11%), and centrum semiovale (17%). While 2TC achieved good curve fitting, it occasionally exhibited instability (relative standard error of *V*_T_ estimation > 10%). When using MA1, among all evaluated t* settings, a t* value of 40 min minimized bias in *V*_T_ estimates (-0.1%) when compared to the 2TC model ($\:\text{M}\text{A}1\:{V}_{\text{T}}\:=\:0.98\:\times\:\:2\text{T}\text{C}\:{V}_{\text{T}}\:+\:0.48,\:{R}^{2}=0.99\:$). *V*_T_ values derived from the MA1 model are summarized in Table 2. The mean *V*_T_ values (mL/cm^3^) ranged from 18.5 ± 3.4 in the centrum semiovale to 40.5 ± 7.0 in the amygdala. A Bland-Altman analysis was performed to assess agreement between 1TC and 2TC, as well as MA1 (t* = 40 min) and 2TC (Supplemental Fig. 1).

### Test-retest variability of *V*_T_

Table 2 presents the TRV, aTRV, and ICC values of *V*_T_ obtained through the ROI-based analysis using the MA1 model. *V*_T_ values demonstrated the volume-weighted mean TRV across regions were minimal (-4.1%) and aTRV less than 10% in all regions except for amygdala (11.1%) and centrum semiovale (11.2%). The volume-weighted mean aTRV across all regions was 4.8%. Supplemental Fig. 2 shows a box-and-whisker plot of TRV. ICC values were generally close to or greater than 0.70, except for the globus pallidus (0.55).

### Effect of scan duration and t* setting on test-retest variability and stable *V*_T_

Table 3 presents the effects of scan duration and t* setting in MA1 on the volume-weighted mean aTRV (panel A) and bias between MA1 and 2TC *V*_T_ estimates (panel B). Table 4 shows the regional effects of scan duration on aTRV of *V*_T_ derived with MA1 for t* value of 40 min. Analysis of the volume-weighted mean values reveals that scan durations of 100, 110, and 120 min produced comparable aTRV across all tested t* values, with deviations within 10% of the 120-min aTRV and minimal bias ranging from − 4% to + 1%. No particular t* setting offered a distinct advantage.

Reducing scan duration moderately increased bias, particularly at early t* settings (t*=10 and 20 min). Nevertheless, a t* of 10 or 20 min with a 90-minute scan duration achieved acceptable bias (within ± 5%) and an aTRV within 15% of the 120-minute aTRV (t*=10 min, bias: -4.8%, aTRV: 4.9%; t*=20 min, bias: -3.2%, aTRV: 5.3%). Regional analysis (Table 2) indicated variations observed across different regions. Specifically, smaller brain regions, such as the amygdala and globus pallidus, required longer scan durations to maintain acceptable bias and comparable aTRV. Optimal t* settings varied by region and scan duration; however, earlier t* values generally resulted in greater underestimation and lower aTRV for a given scan duration (Table 3).

## Discussion

The current study investigated model selection and test-retest reproducibility of [^18^F]Bavarostat binding in the human brain. Moderate bias was observed for 1TC compared to 2TC. To address the large rSE in 2TC *V*_T_ estimates, the MA1 model was applied. Results demonstrated favorable test-retest variability of [^18^F]Bavarostat *V*_T_ with a mean aTRV of 4.8% for a one-month interscan interval. This finding aligns with the previously reported aTRV of 7.7% for a 1-day interval [18]. The consistency across these two studies suggests that [^18^F]Bavarostat exhibits good stability over time. Given its favorable reproducibility, [^18^F]Bavarostat shows potential for future PET studies investigating HDAC6 expression, particularly in the assessment of stress and anxiety disorders. Preclinical studies in rats have revealed decreased [^18^F]Bavarostat uptake in stressed animals, negatively correlating with anxiety levels [24]. These findings indicate potential clinical applications in diagnosing and monitoring stress-related conditions.

The optimal combination of scan duration and t* setting for accurate and reproducible *V*_T_ estimation is dependent on the target brain region and the specific research question. To date, there is no consensus on the criteria for selecting the best combination of scan duration and t* setting. In this study, we used the following parameters: (1) a bias within ± 5% compared to the 2TC model *V*_T_ and (2) an aTRV change within 15% of that observed with a 120-minute scan (for each t* setting). Under these criteria, a 90-minute scan with a t* of 10 or 20 min may be sufficient (Table 3). Importantly, our selection was not based on minimizing aTRV itself, but rather on identifying combinations that produced results comparable to those obtained with the full 120-minute scan.

Careful analysis of the HPLC data for the metabolites was essential due to the small difference in elution times and overlap of metabolite peaks with the parent compound, necessitating meticulous separation during analysis (Fig. 1D). To further explore the influence of potential errors in parent fraction measurement on *V*_T_ estimates, simulations were performed. Noise-free TACs were generated using a population average metabolite-corrected input function and mean kinetic parameters derived from the 2TC model in the insula, the region that provided the most reliable estimates of 2TC microparameters. Subsequently, simulated biased input functions were generated by multiplying the original input function with an exponential decay function (exp(−α* *t*), where *α* = 0.001 (or −0.001)). Using these simulated curves, *V*_T_ was estimated using the MA1 model (t* = 40 min) and the % bias in *V*_T_ values due to the altered metabolite levels was determined. Specifically, for *α* of + 0.001 (or −0.001), i.e., a change of −6% (or + 6%) at 60 min and − 9% (or + 9% at 90 min) in the parent fraction resulted in a 4% increase (or decrease) in the *V*_T_ value. This highlights the importance of meticulous metabolite correction for reliable *V*_T_ quantification in future research.

Additionally, the presence of a lipophilic metabolite relative to the parent compound in Fig. 1D was noted. Uptake of such a metabolite could cause a scan duration-dependent increase in *V*_T_ [25]. Here, *V*_T_ showed minimal increase as a function of scan duration. The volume-weighted mean bias of 2TC *V*_T_ shifted from − 2.5% for a 60-minute scan to -1.7% for a 110-minute scan when compared to the 2TC *V*_T_ from a 120-minute scan, suggesting that the uptake of the lipophilic metabolite had a minor effect on *V*_T_ values. For any region of a scan where the rSE exceeded 10% at any scan duration, all data for that region across all durations were excluded to ensure the same sample size across all scan durations.

Our *V*_T_ values were approximately 23% lower compared to a previous report [18]. After careful examination, we identified several factors that may be contributing to this discrepancy, including differences in ROI definitions, the gray matter masks used for generating TACs, and variations in mean total plasma levels between the studies. Additionally, the HPLC issues discussed earlier and differences in the methodology for generating the input function might also play a role. Further investigation with standardized data processing protocols across studies is needed to fully understand the reasons behind these discrepancies.

This study has some limitations. The relatively small sample size (*n* = 6) necessitates further investigation with a larger cohort. While this sample size is common for PET test-retest studies in human participants ([^18^F]Bavarostat, *n* = 4, 1-day apart [18]; [^18^F]SynVesT-1, *n* = 6, 7 ± 7 days apart [26]; 6-bromo-7-[^11^C]methylpurine, *n* = 6, 20 ± 10 days apart [27]; [^18^F]FPEB, *n* = 9, 6-month apart [28]), a larger sample would provide more robust data. Further studies with more participants would be advantageous to strengthen our observations.

## Conclusions

This study demonstrates promising results for [^18^F]Bavarostat as a PET tracer for HDAC6 imaging. The favorable kinetic modeling characteristics and good test-retest variability with a one-month interscan interval support for research applications investigating HDAC6 expression. potential. However, considerations should be made for certain regions with slightly higher variability and the impact of scan duration on both test-retest results and *V*_T_ estimates. Future studies with larger cohorts might further solidify these findings and explore the optimal scan duration protocol for different brain regions.

**Table 1:** Participant information and PET scan parameters (mean ± SD)

Parameter	Test (*n* = 6)	Retest (*n* = 6)
Age (y)	49 ± 10	
Sex (M/F)	3/3	
Body weight (kg)	80.7 ± 12.3	
Injected dose (MBq)	131 ± 48	150 ± 53
Molar activity at TOI (MBq/nmol)	50 ± 27	84 ± 56
Injected mass (µg)	1.02 ± 0.36	0.80 ± 0.35
Plasma free fraction	0.033 ± 0.010	0.027 ± 0.011

TOI: time of injection

**Table 2:** Distribution volume (*V*_T_) derived from the multilinear analysis 1 (t* = 40 min) and test-retest reproducibility from 120-minute PET data

Regions	*V*_T_ (mL/cm^3^)		TRV (%)		aTRV (%)	ICC
	Test (%COV)	Retest (%COV)	Mean ± SD	Mean		
Amygdala	40.0 (13%)	41.0 (22%)	-1 ± 15	11		0.71
Putamen	33.6 (13%)	34.5 (12%)	-3 ± 4	4		0.94
Ant. Cingulate	32.8 (15%)	34.2 (13%)	-4 ± 7	6		0.86
Insula	33.1 (15%)	34.4 (14%)	-4 ± 4	4		0.93
Hippocampus	32.6 (13%)	34.3 (16%)	-5 ± 8	7		0.82
Cerebellum	31.6 (15%)	34.4 (20%)	-8 ± 9	8		0.74
Globus Pallidus	32.1 (13%)	33.0 (11%)	-3 ± 12	9		0.55
Temporal	32.2 (16%)	33.5 (16%)	-4 ± 3	4		0.95
Caudate	28.7 (12%)	29.3 (14%)	-2 ± 6	4		0.90
Thalamus	28.7 (15%)	29.2 (16%)	-1 ± 3	3		0.98
Frontal	28.3 (14%)	28.8 (13%)	-2 ± 4	3		0.96
Parietal	27.9 (18%)	28.6 (18%)	-2 ± 2	2		0.99
Occipital	25.9 (17%)	27.2 (14%)	-5 ± 4	6		0.93
Post. Cingulate	25.3 (16%)	26.7 (17%)	-5 ± 5	6		0.93
Centrum Semiovale	17.5 (17%)	19.6 (19%)	-11 ± 11	11		0.70

%COV: coefficient of variation (inter-subject variability)

TRV = 100% × (test value − retest value)/[(test value + retest value)/2]. aTRV: absolute value of TRV. ICC: intraclass correlation coefficient

**Table 3:** Timing effects on *V*_T_ results: scan duration and multilinear analysis 1 (MA1) t* setting

**A. Effect on aTRV**							
	**aTRV (%) with different scan duration (min)**						
***t*** *	**60**	**70**	**80**	**90**	**100**	**110**	**120**
**10**	5.2	5.1	5.0	4.9	4.7	4.6	4.5
**20**	5.5	5.8	5.6	5.3	5.0	4.8	4.7
**30**	5.6	6.2	5.8	5.4	5.0	4.7	4.6
**40**	18.8	8.6	6.8	5.9	5.3	4.9	4.8
**50**		10.6	6.8	5.6	5.3	4.9	5.0
**60**			8.2	6.4	5.4	5.0	5.3
**B. Effect on bias between MA1 and 2TC ** ***V***_**T**_ ** estimates**							
	**Bias (%) with different scan duration (min)**						
***t*** *	**60**	**70**	**80**	**90**	**100**	**110**	**120**
**10**	-7.9%	-6.5%	-5.5%	-4.8%	-4.0%	-3.3%	-2.6%
**20**	-5.2%	-4.3%	-3.7%	-3.2%	-2.7%	-2.0%	-1.4%
**30**	-4.1%	-3.3%	-2.8%	-2.5%	-2.0%	-1.3%	-0.7%
**40**	-3.3%	-2.4%	-2.2%	-1.9%	-1.4%	-0.7%	-0.1%
**50**		2.2%	-1.0%	-1.2%	-0.7%	-0.1%	0.6%
**60**			-0.6%	-1.3%	-0.5%	0.4%	1.2%

aTRV: 100% ×|test value − retest value|/[(test value + retest value)/2]

**Table 4:** Regional effect of scan duration on aTRV of *V*_T_ derived with the MA1 model (t* = 40 min)

Regions	aTRV (%) with different scan duration (min)						
	60	70	80	90	100	110	120
Amygdala	1813	39	38	18	15	12	11
Putamen	14	11	7	4	5	4	4
Insula	14	8	8	7	7	6	6
Ant. Cingulate	11	8	10	7	6	5	4
Hippocampus	23	12	10	12	10	8	7
Cerebellum	15	14	10	10	9	9	8
Globus pallidus	43	84	9	12	15	12	9
Temporal	8	6	6	4	4	4	4
Caudate	15	6	6	8	6	4	4
Thalamus	7	5	5	5	3	3	3
Frontal	8	7	6	5	4	4	3
Parietal	7	5	3	3	2	2	2
Occipital	6	5	5	5	5	5	6
Post. Cingulate	19	6	5	6	6	6	6
Centrum semiovale	20	20	14	15	12	13	11

*aTRV, 100% ×|test value − retest value|/[(test value + retest value)/2]

## Electronic supplementary material

Below is the link to the electronic supplementary material.

Supplementary Material 1

## Data Availability

The datasets used and/or analyzed during the current study are available from the corresponding author on reasonable request.
