# Supplementary material for: Assessment of test-retest reproducibility by [18F]Bavarostat for PET imaging of HDAC6
Source: EJNMMI Res. 2025 Jun 21;15:76. doi: 10.1186/s13550-025-01268-w (PMC12182542; doi:10.1186/s13550-025-01268-w)
Supplement: Supplementary file 1 — Supplementary Material 1 [file 13550_2025_1268_MOESM1_ESM.pdf]

Assessment of Test-retest Reproducibility by [^{18}F]Bavarostat for PET imaging of HDAC6

Mika Naganawa[1], Ming-Qiang Zheng[1], Jean-Dominique Gallezot[1], Robin Bonomi[1], Jiwei Gu[1], Hong Gao[1], Swanee Jacutin-Porte[1], Nabeel B. Nabulsi[1], Michel Koole[2], Koen Van Laere[2], David Matuskey[1], Yiyun Huang[1], Richard E. Carson[1]

[1]Yale PET Center, Yale School of Medicine, New Haven, USA
[2]Nuclear Medicine and Molecular Imaging, Department of Imaging & Pathology, KU Leuven, Leuven, Belgium

Email of Corresponding Author: mika.naganawa@yale.edu

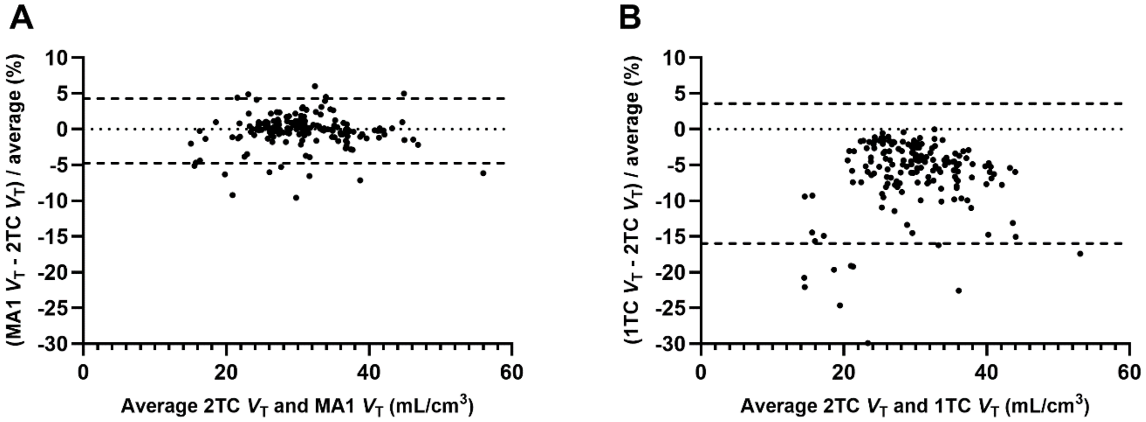

Supplemental Figure 1: Bland-Altman plots assessing the agreement of distribution volume (V_T) estimates between (A) the multilinear analysis 1 (MA1) and the two-tissue compartment (2TC) model, and (B) the one-tissue compartment (1TC) model and the 2TC model. Data points where the 2TC V_T values had a high %SE (>10%) were excluded.

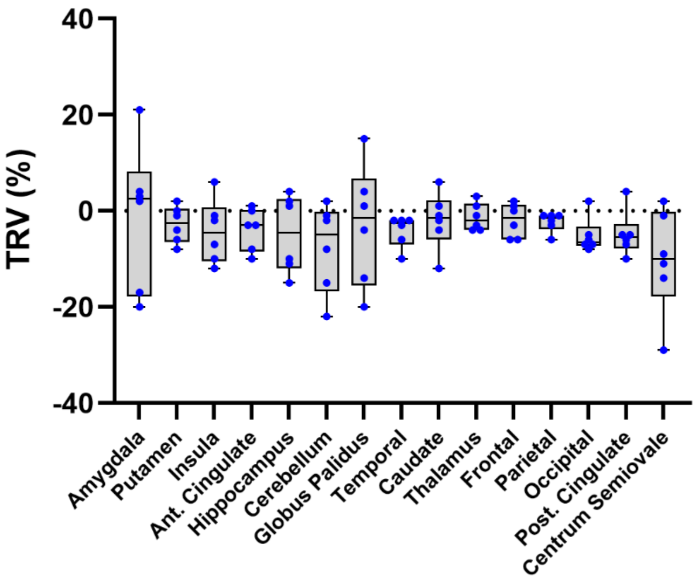

Supplemental Figure 2: Box-and-whisker plots of test-retest variability (TRV) of all participants. Each data point represents the TRV for an individual participant (blue), with one participant's TRV (from 223-day scan interval) highlighted in red.
